# Five Years of Antimalarial Resistance Marker Surveillance in Gaza Province, Mozambique, Following Artemisinin-Based Combination Therapy Roll Out

**DOI:** 10.1371/journal.pone.0025992

**Published:** 2011-10-14

**Authors:** Jaishree Raman, Katya Mauff, Pedro Muianga, Abdul Mussa, Rajendra Maharaj, Karen I. Barnes

**Affiliations:** 1 Malaria Research Programme, Medical Research Council, Durban, South Africa; 2 Division of Clinical Pharmacology, Department of Medicine, University of Cape Town, Cape Town, South Africa; 3 Department of Statistical Science, University of Cape Town, Cape Town, South Africa; 4 Gaza Province Directorate of Health, Xai Xai, Mozambique; 5 National Malaria Control Programme, Maputo City, Mozambique; Université Pierre et Marie Curie, France

## Abstract

Antimalarial drug resistance is a major obstacle to malaria control and eventual elimination. The routine surveillance for molecular marker of resistance is an efficient way to assess drug efficacy, which remains feasible in areas where malaria control interventions have succeeded in substantially reducing malaria transmission. Community based asexual parasite prevalence surveys were conducted annually in sentinel sites in Gaza Province, Mozambique from 2006 until 2010, before, during and after antimalarial policy changes to artesunate plus sulfadoxine-pyrimethamine in 2006 and to artemether-lumefantrine in 2008. Genetic analysis of *dhfr*, *dhps*, *crt*, and *mdr1* resistant genes was conducted on 3 331 (14.4%) *Plasmodium falciparum* PCR positive samples collected over the study period from 23 229 children aged 2 to 15 years. The quintuple *dhfr/dhps* mutation associated with sulfadoxine-pyrimethamine resistance increased from 56.2% at baseline to 75.8% by 2010. At baseline the *crt*76T and *mdr1*86Y mutants were approaching fixation, 96.1% and 74.7%, respectively. Following the deployment of artemisinin-based combination therapy, prevalence of both these chloroquine-resistance markers began declining, reaching 32.4% and 30.9%, respectively, by 2010. All samples analysed over the 5-year period possessed a single copy of the *mdr1* gene. The high and increasing prevalence of the quintuple mutation supports the change in drug policy from artesunate plus sulfadoxine-pyrimethamine to artemether-lumefantrine in Mozambique. As chloroquine related drug pressure decreased in the region, so did the molecular markers associated with chloroquine resistance (*crt*76T and *mdr1*86Y). However, this reversion to the wild-type *mdr186N* predisposes parasites towards developing lumefantrine resistance. Close monitoring of artemether-lumefantrine efficacy is therefore essential, particularly given the high drug pressure within the region where most countries now use artemether-lumefantrine as first line treatment.

## Introduction

Despite being a readily preventable and treatable disease, malaria remains a major global health burden [1]. One of the main factors contributing to this sustained burden is the emergence and spread of antimalarial drug resistance [2]. In an attempt to ensure effective treatment as well as delay the emergence of antimalarial resistance the WHO recommended that combination therapy using artemisinin derivatives, replace all monotherapies as first line treatment for uncomplicated malaria [3]. Artemisinin-based combination therapies (ACTs) rapidly decrease parasite load, increase cure rate, are effective against gametocytes (the source of onward transmission of malaria) and have the potential to delay the emergence and spread of antimalarial drug resistance [4], [5]. However, recent studies have shown that the spread of resistance markers is not always impeded by ACT implementation, particularly if resistance to the partner drug had previously been established within the region [6], [7].

Malaria is major cause of morbidity and mortality in Mozambique, with approximately 6 million cases annually [1]. The Lubombo Spatial Development Initiative (LSDI) malaria programme using community based indoor residual spraying (IRS) together with effective malaria treatment was highly successful in Maputo Province, Mozambique, where malaria prevalence in children aged 2–14 years declined from between 64 to 87% at baseline to below 15% after 7 years of intensive malaria control [7], [8], [9]. Based on the advances made in Maputo Province, the malaria programme was extended into neighbouring Gaza Province in 2006. The intervention focused on community based IRS as the national Mozambican malaria treatment policy had already been changed from chloroquine monotherapy to an artemisinin-based combination therapy.

In Mozambique, first line treatment for uncomplicated malaria changed from chloroquine to sulfadoxine-pyrimethamine (SP) plus amodiaquine in 2004 and then to the artemisinin-based combination (ACT), artesunate plus SP in 2006 [7], [10]. Following the phased pilot implementation of the artesunate plus SP in Maputo Province between 2004 and 2006, molecular markers associated with SP resistance increased dramatically [7], raising concern over the effective therapeutic lifespan of artesunate plus SP. This contributed to a further change in national malaria treatment policy in 2008 when the fixed dose combination of artemether-lumefantrine became the recommended first line treatment for uncomplicated malaria. As is not unusual outside of a research programme, there was some delay in the implementation of these changes in national malaria treatment policies in Gaza Province, where full deployment took 1–2 years.

While *in vivo* clinical trials are considered the gold standard for measuring drug efficacy, they are very expensive, time consuming, labour intensive and require a relatively high number of malaria cases presenting to the study site. In areas where malaria control measures have succeeded in reducing malaria transmission intensity substantially, a more feasible manner to monitor drug efficacy is the routine surveillance for molecular markers associated with treatment failure. We report on the prevalence of molecular markers associated with lumefantrine, chloroquine and SP resistance in the five years since the introduction of ACTs (artesunate plus SP and then artemether-lumefantrine) in Gaza Province, Mozambique.

## Materials and Methods

### Study area

**Figure 1:**
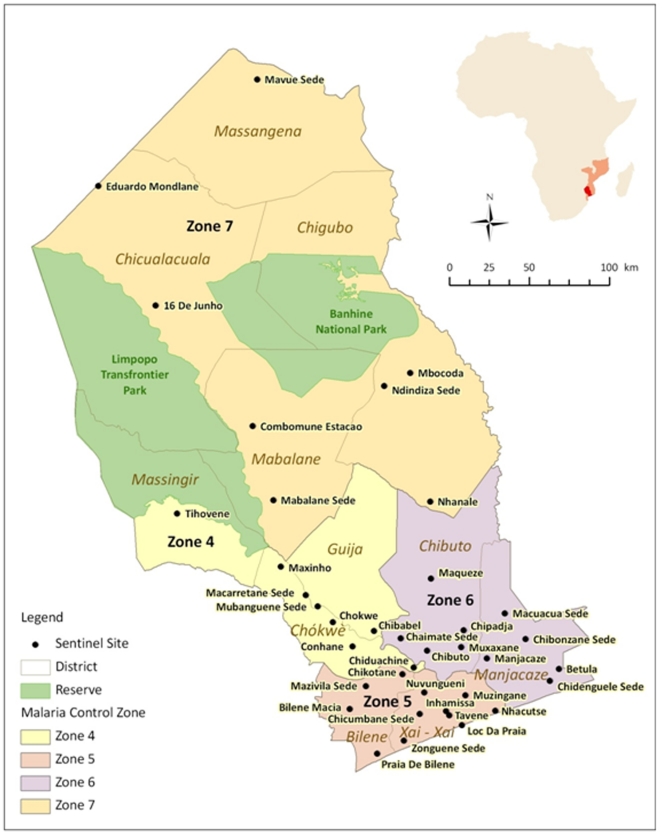
Sentinel Sites and Zones in Gaza Province, Southern Mozambique.

The study was conducted at 38 sentinel sites across Gaza Province, southern Mozambique (Figure 1) during annual community-based cross sectional malaria prevalence surveys from 2006 until 2010. The province encompasses an area of 75 709 km^2^, with an approximate population of 1.5 million, and for the study the 7 districts in Gaza Province were grouped into 4 geographic zones. Zones differed in terms of their population density and level of economic development, with Zone 5 where the provincial capital is situated being the most developed and Zone 7 having the lowest population density and the least economic development. Malaria is endemic to the region with transmission peaking during the rainy season from October to April. The majority of the reported malaria cases are caused by *Plasmodium falciparum*.

### Study population and blood sample collection

Finger prick filter paper blood samples were collected during the annual malaria prevalence surveys from 120 to 150 children (aged between 2 and ≤15 years) at each of the 30 sentinel sites spread across Zones 4, 5 and 6 in Gaza Province, Mozambique (Figure 1), from 2006 to 2007. In 2008 Zone 7 with 8 additional sentinel sites was added to the survey area (Figure 1). Capillary blood samples, blotted on filter paper (3MM Whatman filter paper, Merck Laboratory Supplies (Pty) Ltd., Durban, South Africa) were air dried and then individually stored at room temperature in zip-lock packets containing desiccant. Blood samples were only taken after informed consent from a parent/guardian had been obtained.

### Sample preparation and analysis

Parasite DNA was extracted from the blood spots of participants found to be rapid test positive (ICT™, Global Diagnostics, Cape Town, South Africa, SD Bioline, SD, Korea) using the Chelex method [11]. Once a sample was confirmed as *P. falciparum* positive by qPCR [12], polymorphism analysis of dihyrofolate reductase (*dhfr*), dihydropteroate synthetase (*dhps*), chloroquine resistance transporter *(crt)* and multidrug resistance 1 (*mdr1*) genes was conducted. Primers, PCR amplification conditions and restriction endonucleases used to detect polymorphisms in the *dhfr* (codons *dhfr*N51I, *dhfr*C59R, *dhfr*S108N, *dhfr*I164L), *dhps* (codons *dhps*S436A, *dhps*A437G, *dhps*K540E and *dhps*A581G), *mdr1* (codon *mdr1N*86Y) and *crt* (codon *crt*K76T) genes have been described previously [13], [14], [15]. Digestion products separated on a 2% agarose gel using electrophoresis were visualised and photographed using a MiniBIS™ documentation system (BioSystematica, United Kingdom). Codons were classified as either pure sensitive, pure mutant or mixed (both mutant and sensitive genotypes present in an individual sample). Genotyping was run in duplicate, with a third assay being performed on any discordant results. When calculating overall prevalence of infections with mutant genotypes, codons with mixed genotypes were grouped with pure mutant codons. Copy number of the *mdr1* gene was assessed using the qPCR method, primers, probes and qPCR cycling conditions previously described by Price et al [16]. Every qPCR run contained two reference DNA samples from D10 and Fac8 clones, having an *mdr1* copy number of 1 and 3 respectively as well as a no template control.

### Statistical analysis

Statistical analysis was performed using Stata 11.0 (Stata Corporation, College Station, Texas). Univariate analysis and multiple variable logistic regression were carried out to determine whether any of the prospectively defined factors (namely age, gender, fever, sentinel site specific asexual parasite prevalence, rural vs peri-urban sentinel site, zone, and study year) were significantly associated with mutation prevalence. Statistical inference took account of within-sentinel site correlations of mutational markers and asexual parasite prevalence; analyses were weighted for the number of PCR positive patients per site using inverse proportional weights. Confidence limits were set at 95%.

### Ethic Statement

Ethical approval for this study was obtained from the South African Medical Research Council and the Gaza Provincial Directorate of Health, Mozambique. Blood samples were only taken if full informed verbal consent from a parent/guardian had been obtained. The researchers involved in this study, with ethical approval from South African Medical Research Council and the Mozambican Ministry of Health opted to obtain of verbal consent for sample collections, for the following reasons:

1. Prior to sample collection occurring, awareness campaigns detailing the purpose, date, time and venue of the prevalence surveys were conducted by community health workers at the sentinel sites and
2. During sample collection, the purpose of the survey was once again explained to the parent/guardian by survey staff on a one to one basis.

The homestead GPS co-ordinates of each parent/guardian approached to participate in the survey was recorded and a note was made of the parents/guardians that declined to participate in the survey. Less than 10% of the parents/guardians approached declined to give consent.

Children testing positive for malaria were referred to the closest health facility for appropriate treatment.

## Results

**Figure 2:**
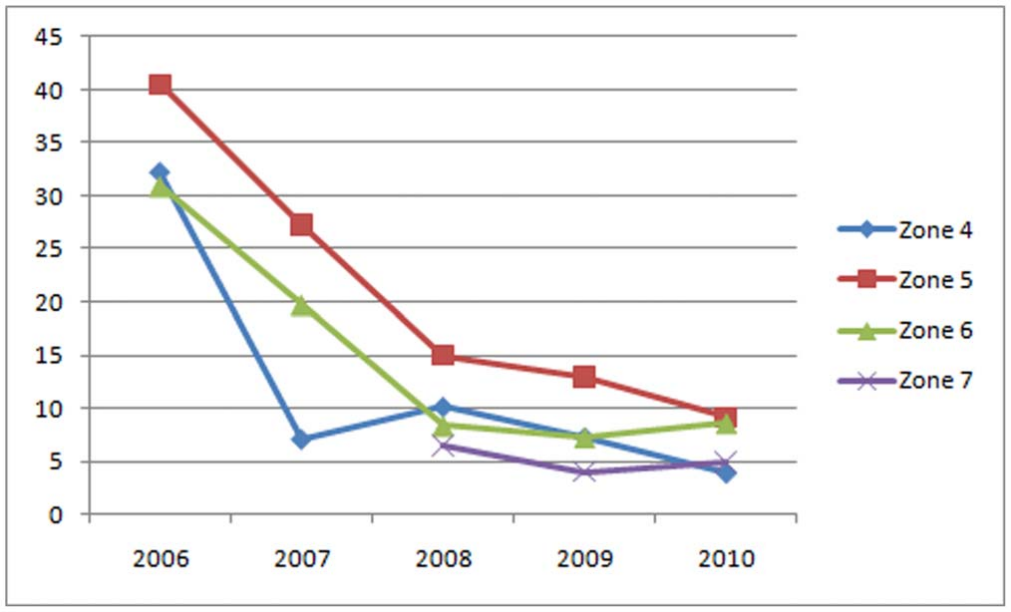
Asexual Parasite Prevalence (%) in Gaza Province by Zone and Year.

Of the 23 229 children surveyed over the five study years, 4 755 (21%) were rapid test positive for *P. falciparum*. Filter paper blood samples were collected from 4 440 (93%) rapid test malaria positive subjects, of which 3 333 (75.1%) were confirmed *P. falciparum* positive by qPCR. The *falciparum* positive samples were obtained from children with a median age of 6 (IQR 4–9) years, of which 48.7% were female and 6.3% febrile (auxiliary temperature ≥37.5°C). The median age of malaria infected children increased from 6 (IQR 4–9) years in 2006 to 7 (IQR 5–10) years by 2010. In 2006 the median PCR confirmed asexual parasite prevalence in Gaza Province was 28% declining to 4% by 2010 (Figure 2). Baseline asexual parasite prevalence varied considerably among the sentinel sites (range 5.9 to 78.7% in 2006 and 0.0 to 45.0% in 2010), as did the rate of decrease in parasite prevalence.

Mutant *dhfr*164L and *dhps*581G alleles were not detected in any of the samples analysed over the study period. All samples analysed had a single copy of the *mdr1* gene. Polymorphisms at codon *dhps*436 were extremely rare occurring in 1.4% (48/3 331) of the samples tested and were only observed in samples collected in 2006. Mixed *dhfr*, *dhps*, *crt*76 and *mdr1*86 alleles were detected in 14.7% (155/3 331), 43.8% (1460/3 331), 17.5% (386/2 205) and 47.1% (1521/3 233) of the samples analysed, respectively.

**Figure 3:**
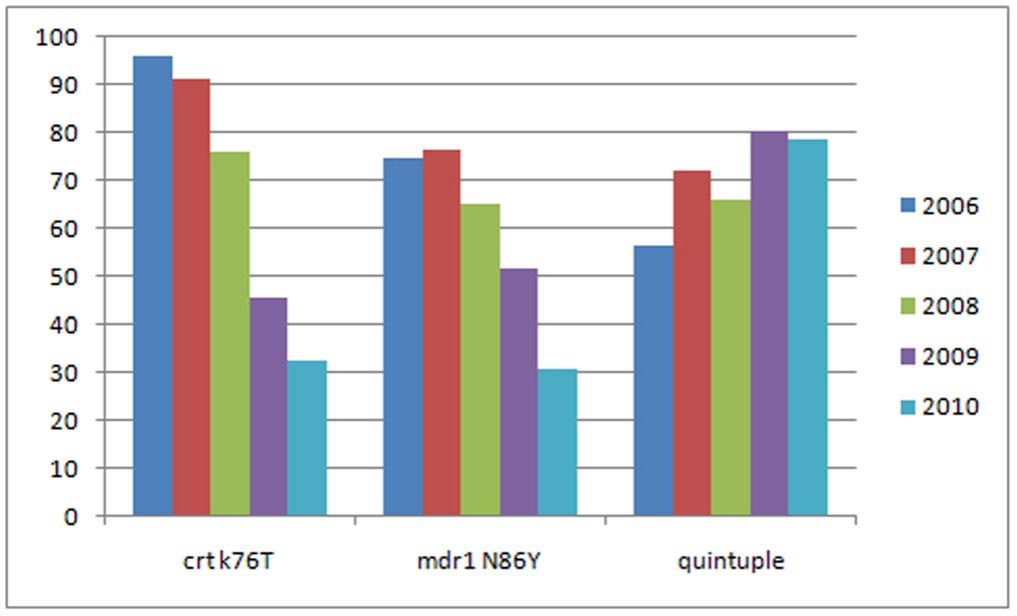
Prevalence of quintuple, *crt*76Tand *mdr1*86Y mutations (%) in Gaza Province by year.

The *dhfr* triple haplotype (codons *dhfr*51I, *dhfr*59R and *dhfr*108N) was close to fixation (98.1%, 1 235/1 259) at baseline and remained unchanged following the roll out of ACTs within the region (OR: 1.01; 95% CI: 0.76–1.34; *P* = 0.950). Given the fixation of the *dhfr* triple mutation in the population, prevalence of parasites carrying the ‘quintuple’ allele (presence of both the *dhfr* triple *and dhps* double mutations), was very similar to the *dhps* double mutation prevalence (codons *dhps*437G and *dhps*540E). At baseline 56.2% (708/1 259) of the parasites analysed carried the quintupl*e* mutation, which increased to 78.5% (314/400) by 2010 (Table 1, Figure 3). Although quintuple mutation prevalence varied considerably between the different Zones at baseline (37.4% in Zone 4, 67.5% in Zone 5, 54.3% in Zone 6 and 31.3% in Zone 7), it increased markedly each year across all Zones over the 5-year study period (OR: 1.21 per year; 95% CI: 1.02–1.46; *P* = 0.034).

**Table 1:** Quintuple, *crt*76T and *mdr*86Y mutation prevalence (%) by Zone and Year.

Year	Zone	Mutation Prevalence		
		Quintuple	*crt*76T	*mdr1*86Y
**2006**	Zone 4	37.5% (116/309)	96.5% (136/141)	79.5% (244/307)
**2007**		36.5% (19/52)	97.4% (37/38)	78.4% (40/51)
**2008**		83.2% (84/101)	88.2% (82/93)	82.8% (82/99)
**2009**		82.8% (77/93)	62.8% (32/51)	53.5% (46/86)
**2010**		68.2% (30/44)	19.2% (5/26)	48.8% (21/43)
**2006**	Zone 5	67.5% (390/578)	96.2% (332/345)	74.9% (427/570)
**2007**		83.3% (340/408)	93.5% (346/371)	78.2% (315/403)
**2008**		65.1% (142/218)	82.4% (159/193)	71.1% (155/218)
**2009**		87.1% (210/241)	41.8 (41/98)	55.9% (124/222)
**2010**		89.6% (147/164)	35.5% (50/141)	30.9% (47/152)
**2006**	Zone 6	54.3% (202/372)	95.5% (148/155)	70.3% (260/370)
**2007**		59.9% (139/232)	86.6% (174/201)	72.7% (162/223)
**2008**		73.2% (79/108)	82.2% (60/73)	53.1% (51/96)
**2009**		75.9% (82/108)	55.0% (22/40)	52.5% (53/101)
**2010**		83.85% (109/130)	37.3% (38/102)	25.8% (32/124)
**2006**	Zone 7	-	-	-
**2007**		-	-	-
**2008**		31.3% (20/64)	30.0% (18/60)	33.3% (21/63)
**2009**		51.1% (24/47)	20.0% (7/35)	26.1% (12/46)
**2010**		45.2% (28/62)	17.5% (7/40)	28.3% (17/60)

After adjusting for survey year and zone, multiple logistic regression analysis confirmed that quintuple mutation prevalence was independently negatively associated with age (OR: 0.89 per year of age; 95% CI: 0.85–0.93; *P*<0.0001), and rural vs. peri urban sentinel sites (OR: 0.59; 95% CI: 0.43–0.79; *P* = 0.001). A slight positive association between quintuple mutation prevalence and sentinel site specific asexual parasite prevalence was found (OR: 1.01; 95% CI: 1.005–1.02; *P* = 0.001) in the logistic model (Table 2). None of the other pre-defined explanatory variables were found to be associated with quintuple mutation prevalence.

**Table 2:** Factors associated with quintuple, *crt*76T and *mdr1*86Y mutation prevalence in Gaza Province between 2006 and 2010 (within site correlations are taken into account in the estimation of confidence intervals).

Covariate	Quintuple Mutation			*crt*K76T			*mdr1*N86Y		
	OR	95% CI	*P* value	OR	95% CI	*P* value	OR	95% CI	*P* value
**2006**	1			1			1		
**2007**	2.50	1.73–3.62	<0.0001	0.36	0.15–0.86	0.022	1.31	0.91–1.90	0.142
**2008**	2.48	1.35–4.55	0.004	0.13	0.05–0.31	<0.0001	0.87	0.53–1.41	0.549
**2009**	9.17	4.44–18.93	<0.0001	0.02	0.01–0.06	<0.0001	0.26	0.14–0.46	<0.0001
**2010**	5.92	3.30–10.61	<0.0001	0.02	0.01–0.05	<0.0001	0.33	0.20–0.55	<0.0001
**Age (years)**	0.89	0.85–0.933	<0.0001	1.13	1.02–1.26	0.020	1.05	1.01–1.10	0.015
**Asexual Parasite Prevalence (%)**	1.01	1.01–1.02	0.001	0.99	0.97–1.01	0.340	1.00	1.00–1.01	0.292
**Rural vs Peri Urban Sentinel Site**	0.59	043–0.79	0.001	2.25	1.13–4.48	0.022	0.92	0.67–1.27	0.592
**Zone 4**	1			1			1		
**Zone 5**	2.32	1.50–3.58	<0.0001	1.05	0.55–2.01	0.876	0.73	0.52–1.04	0.079
**Zone 6**	1.48	0.94–2.33	0.091	0.61	0.29–1.30	0.196	0.60	0.42–0.87	0.008
**Zone 7**	0.51	0.29–0.90	0.022	0.22	0.07–0.66	0.009	0.36	0.17–0.80	0.014

At baseline the *crt*76T mutant allele was approaching saturation within the population (96.1%, 616/641). However following the replacement of CQ with combination treatments in Gaza Province, prevalence of this mutation declined annually (OR: 0.33 per year; 95% CI: 0.26–0.42; *P*<0.0001, Figure 3, Table 1) dropping to 32.36% (100/309) by 2010.

A positive association between *crt*76T mutation prevalence and rural vs. peri-urban sentinel sites (OR: 2.25; 95% CI: 1.13–4.47; *P* = 0.022) as well as age (OR: 1.13 per year of age; 95% CI: 1.02–1.26; *P* = 0.020) was noted in the multiple logistic regression analysis, after adjusting for survey year, zone and site specific asexual parasite prevalence. This model showed no association between *crt*76T mutation prevalence and sentinel site specific parasite prevalence (OR: 0.99; 95% CI: 0.97–1.01; *P* = 0.340), nor any of the other pre-defined explanatory variables (Table 2).

**Figure 4:**
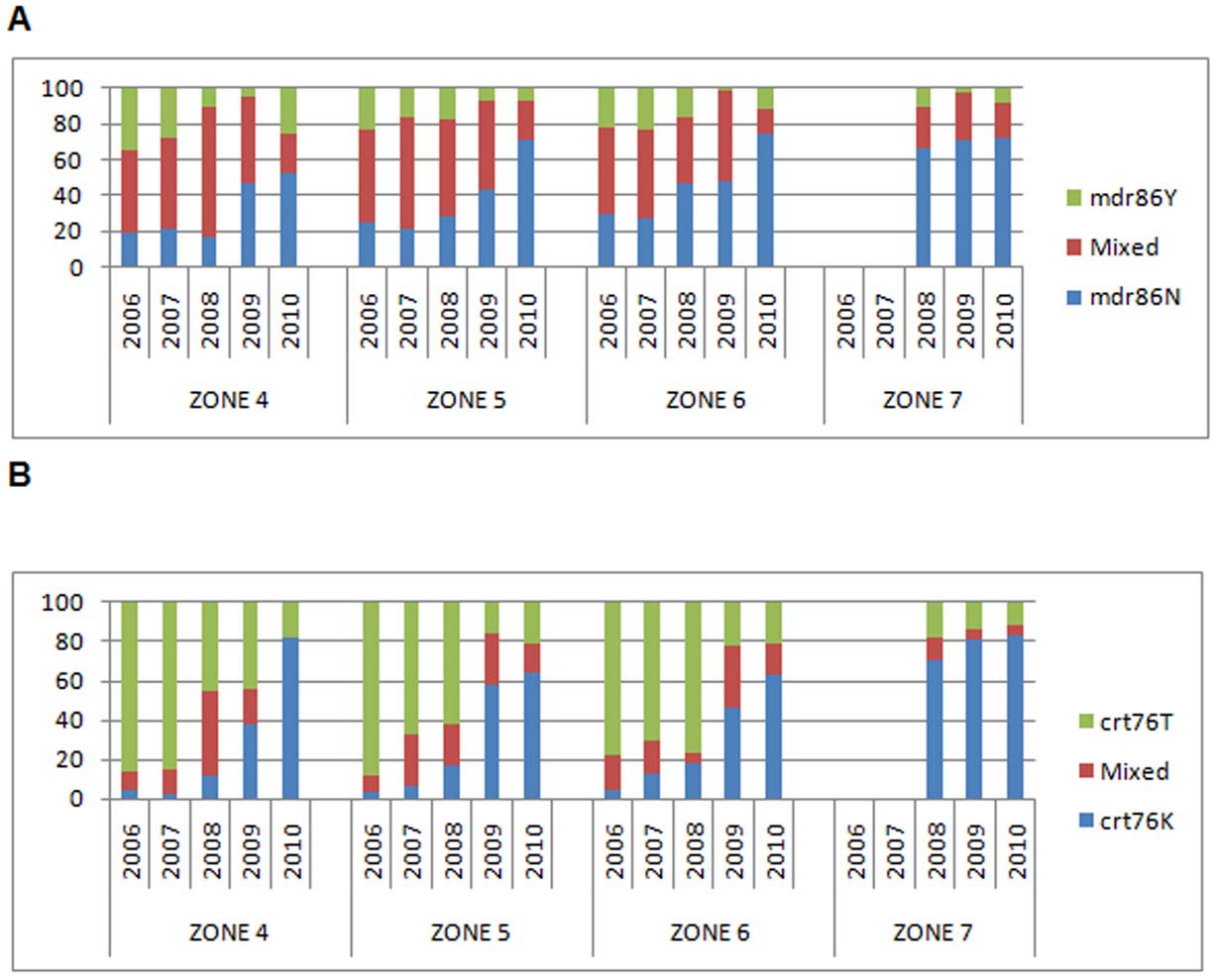
Prevalence of pure wild, pure mutant and mixed *crt*76 and *mdr1*86 alleles (%) in Gaza Province by Year and Zone.

Most the parasites analysed at baseline carried the mutant *mdr*86Y haplotype (74.7%, 931/1 247). Prevalence of this mutation remained unchanged from baseline in 2007 (OR: 1.31; 95% CI: 0.91–1.90; *P* = 0.142), but began declining thereafter (OR: 0.63 per year; 95% CI: 0.55–0.71; *P*<0.0001, Figure 3, Table 1), reaching 30.9% (117/379) by 2010. Only age (OR: 1.05 per year of age; 95% CI: 1.01–1.09; *P* = 0.015) was shown to be associated with *mdr*86Y mutation prevalence in the multiple logistic regression model, after controlling for survey year, zone, rural vs. urban site and site specific asexual prevalence. None of these variables appeared to influence *mdr*86Y mutation prevalence (Table 2). By 2010 the number of samples carrying mixed haplotypes at either codon *crt*76 or *mdr*86 had decreased markedly (Figure 4), reflecting a decrease in transmission rate.

## Discussion

The success of an integrated malaria initiative is dependent upon each component of the initiative functioning optimally in its own right. One of the biggest challenges for most control programmes is limiting the emergence and spread of antimalarial drug resistance [17]. Thus the close monitoring of drug efficacy is vital to allow for the timely implementation of changes in malaria treatment policy. Changes in antimalarial drug policy in Mozambique have had a significant effect on the resistant marker prevalence in Gaza Province.

Following the implementation of an integrated malaria control programme in Gaza Province, which included community based IRS operations, definitive diagnosis using rapid diagnostic test kits, effective treatment with ACTs and Intermittent Preventative Treatment (IPT) using SP, PCR-confirmed *falciparum* malaria prevalence has declined significantly from a mean of 30% pre-intervention to below 15% after five years of control. The accumulation of mutations in the *dhfr* and *dhps* genes, the targets of sulphadoxine-pyrimethamine (SP), are associated with SP treatment failure [18]. Three mutations in the *dhfr* gene (codons *dhfr*108N, *dhfr*51I and *dhfr*59R), known as the *dhfr* triple predict pyrimethamine drug failure while two mutations in *dhps* gene (codons *dhps*437G and *dhps*540E), called the *dhps* double are strongly associated with sulphadoxine treatment failure. Mutations in the *dhfr* and *dhps* genes usually occur a stepwise fashion; however in East and Southern Africa these mutations have spread via selective sweeps [18], [19]. Parasites carrying all five mutations, commonly called the quintuple mutation are associated with SP treatment failure in southern and East Africa [18]. At baseline the *dhfr* triple mutation was at fixation (98%) in the population, with over 50% of the parasites in the regions carrying quintuple mutations.

Allen and colleagues showed the presence of the quintuple mutation resulted in a 3-fold increased risk of treatment failure in neighbouring Maputo Province, after adjusting for treatment arm (SP monotherapy versus artesunate plus SP), age and temperature [20]. As seen in Maputo Province [7], the roll out of the artesunate plus SP did not halt the spread of the SP resistance parasites, with 70% of all parasites analysed in 2010 carrying the quintuple mutation. Despite these high levels of SP resistance, the *dhfr*I164L mutation associated with high pyrimethamine resistant parasite has not been detected in the region. None the less, the rapid increase in the prevalence of the quintuple mutation supports the decision taken by the Mozambican Ministry of Health in 2008 to replace artesunate plus SP with artemether-lumefantrine. The sustained high quintuple mutation prevalence in Gaza Province is not unexpected when three facts are taken into consideration. First, SP is still being used for intermittent preventative treatment in pregnancy, second, although artemether-lumefantrine was adopted as first line treatment in 2008, complete deployment of the drug in Gaza Province was only achieved by 2010 and thirdly, the use of other antifolate-sulfonamide combinations like cotrimoxazole as prophylaxis against opportunistic infection in HIV/AIDS patients [21] may contribute to cross-resistance. Other countries, including India, that have selected artesunate plus SP as first line therapy should monitor the efficacy of this treatment closely, given the unusually short useful therapeutic life of SP, even when used in combination with artesunate.

In contrast to SP resistance makers, the *mdr1*N86Y mutation associated with chloroquine resistance (but lumefantrine sensitivity) declined from 75% at baseline to 31% in 2010. This shift is likely to be a result of removal of chloroquine drug pressure following the introduction of ACTs, as seen in Malawi [22]. Mutations in the *P falciparium mdr1* gene appear to modulate the effectiveness of chloroquine, amodiaquine, mefloquine and lumefantrine [23]. Of the identified *mdr1* mutations, the *mdr1*86Y mutation is most commonly associated with chloroquine and amodiaquine resistance and sensitivity to mefloquine and lumefantrine [23], [24], [25], [26]. As an increase in the *mdr1*86N allele prevalence has been suggested as the first step to lumefantrine tolerance [27], our results suggest the need for close monitoring of artemether-lumefantrine efficacy in Mozambique and its neighbouring countries.

The increase in *mdr1* copy number associated with *in vitro* lumefantrine resistance in South-east Asia [28] was not detected in any of the samples analysed in this study. Studies in South East Asia have shown that the amplification of *mdr1* gene is associated with mefloquine [16] and possibly lumefantrine resistance [28]. Our findings support the suggestion that *mdr1* amplification is rare in Africa [23]. This lack of *mdr1* amplification may be a consequence of the high use of chloroquine in Africa, the absence of mefloquine drug pressure and the relatively short duration of widespread artemether-lumefantrine use [29].

Multivariable analysis indicated that SP drug pressure was greater in peri-urban areas and younger children, while chloroquine use appears to have been sustained longer in rural areas and older children. The negative association between quintuple mutation prevalence and age but positive association between the *crt*76T mutation prevalence and age could be an indication of variable treatment seeking behaviours within Gaza Province; with younger children diagnosed and treated at health facilities with artesunate plus SP, while older children may be diagnosed and treated at home with chloroquine. Our finding of a small but significant positive association between quintuple mutation prevalence and sentinel site specific asexual parasite prevalence contrasts with historical evidence that drug resistance generally arises and spreads most rapidly in areas of low intensity malaria transmission, where lack of immunity would be expected to increase treatment seeking and facilitate the survival of resistant parasites. One plausible explanation of our unexpected finding would be that higher drug resistance increases malaria transmission in these sentinel sites, by increasing gametocyte carriage in both the primary [30] and recrudescent infections [16].

The molecular make up of malaria parasites responds rapidly to changes to drug pressure, making the continued monitoring for polymorphisms associated with drug resistance essential [31]. Despite limited use of artemether-lumefantrine in Gaza Province, markers associated with resistance/tolerance to lumefantrine are already present in the population, a consequence of reduced chloroquine drug pressure. This is particularly concerning as artemether-lumefantrine has become first line treatment in most southern African countries, and increased drug pressure can exert an influence on drug efficacy in neighbouring countries as previously seen in Mozambique [9] and Swaziland [32].

Results from this study support the decision taken to replace artesunate plus SP with artemether-lumefantrine, given the quintuple mutation nearing fixation. The high prevalence of this mutation also questions the useful therapeutic life of SP monotherapy for IPT in this region. A recent Tanzanian study [33] has shown while IPT does not confer any benefit in an area of widespread resistance, it may increase the odds of fetal anemia. We therefore recommend the re-evaluation of IPT using SP in Mozambique. Additionally, in light of the presence of molecular makers associated with lumefantrine tolerance/resistance in the population, we strongly support the continued routine surveillance for antimalarial drug resistance markers to ensure the recent gains made by the malaria control programme in Gaza Province are sustained.
